# Remote photobiomodulation targeted at the abdomen or legs provides effective neuroprotection against parkinsonian MPTP insult

**DOI:** 10.1111/ejn.15973

**Published:** 2023-04-06

**Authors:** Luke C. Gordon, Kristy L. Martin, Napoleon Torres, Alim‐Louis Benabid, John Mitrofanis, Jonathan Stone, Cecile Moro, Daniel M. Johnstone

**Affiliations:** ^1^ School of Medical Sciences University of Sydney Sydney New South Wales Australia; ^2^ Univ. Grenoble Alpes, CEA, LETI, Clinatec 38000 Grenoble France; ^3^ School of Biomedical Sciences & Pharmacy University of Newcastle Callaghan New South Wales Australia

**Keywords:** animal model, MPTP, neuroprotection, Parkinson's disease, photobiomodulation, remote

## Abstract

Photobiomodulation (PBM)—the irradiation of tissue with low‐intensity light—mitigates neuropathology in rodent models of Parkinson's disease (PD) when targeted at the head (‘transcranial PBM’). In humans, however, attenuation of light energy by the scalp and skull necessitates a different approach. We have reported that targeting PBM at the body also protects the brain by a mechanism that spreads from the irradiated tissue (‘remote PBM’), although the optimal peripheral tissue target for remote PBM is currently unclear. This study compared the neuroprotective efficacy of remote PBM targeting the abdomen or leg with transcranial PBM, in mouse and non‐human primate models of PD. In a pilot study, the neurotoxin MPTP was used to induce PD in non‐human primates; PBM (670 nm, 50 mW/cm^2^, 6 min/day) of the abdomen (*n* = 1) was associated with fewer clinical signs and more surviving midbrain dopaminergic cells relative to MPTP‐injected non‐human primates not treated with PBM. Validation studies in MPTP‐injected mice (*n* = 10 per group) revealed a significant rescue of midbrain dopaminergic cells in mice receiving PBM to the abdomen (~80%, *p* < .0001) or legs (~80%, *p* < .0001), with comparable rescue of axonal terminals in the striatum. Strikingly, this degree of neuroprotection was at least as, if not more, pronounced than that achieved with transcranial PBM. These findings confirm that remote PBM provides neuroprotection against MPTP‐induced destruction of the key circuitry underlying PD, with both the abdomen and legs serving as viable remote targets. This should provide the impetus for a comprehensive investigation of remote PBM‐induced neuroprotection in other models of PD and, ultimately, human patients.

## Graphical abstract

A pilot study in non‐human primates provided initial evidence that remote photobiomodulation, particularly when targeted at the abdomen, can mitigate clinical signs of Parkinson's disease in an MPTP neurotoxin model. Validation studies in a larger cohort of mice confirmed that remote PBM of the abdomen and legs mitigates PD‐like damage to the nigrostriatal pathway. These data support further development of remote PBM as a neuroprotective intervention.

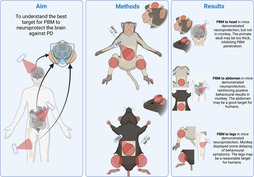

**Abbreviations**
CPu: caudate–putamen complex
MPTP: 1‐methyl‐4‐phenyl‐1,2,3,6‐tetrahydropyridine
PBM: photobiomodulation
PBS: phosphate‐buffered saline
PD: Parkinson's disease
SNc: substantia nigra pars compacta
TH: tyrosine hydroxylase

## 1 INTRODUCTION

Studies of rodent models, beginning with the work of Eells et al. (2003) on retina, have consistently demonstrated the neuroprotective actions of photobiomodulation (PBM)—the irradiation of tissue with low‐energy red to near‐infrared light (600–1100 nm) (Salehpour et al., 2018). In the context of Parkinson's disease (PD), we have shown that PBM with 670‐ or 810‐nm light, directed transcranially, mitigates dopaminergic cell loss in mice exposed to the neurotoxin MPTP (1‐methyl‐4‐phenyl‐1,2,3,6‐tetrahydropyridine) (Moro et al., 2013; Peoples et al., 2012; Reinhart et al., 2015, 2017; Shaw et al., 2010), partially restores normal neuronal activity in the basal ganglia (Shaw et al., 2012) and improves motor behaviour (Moro et al., 2013; Reinhart et al., 2015). In addition to providing neuroprotection against acute insults, we have demonstrated that regular transcranial PBM also protects against chronic neuropathology in the K369I *tau* transgenic model mouse, which over several months develops severe motor abnormalities and dopaminergic cell loss consistent with parkinsonism (Purushothuman et al., 2013). Other investigators have observed similar neuroprotective effects in PD models using other species, such as rats (Oueslati et al., 2015) and flies (Vos et al., 2013), as reviewed elsewhere (Johnstone et al., 2021; Petrucco et al., 2020). In addition, clinical trials assessing the safety profile of transcranial or multi‐site PBM have established that, when applied within the correct dose window, the treatment is safe and not associated with an increase in adverse events (Blivet et al., 2022; Cassano et al., 2022; McGee et al., 2022). Histological observations in non‐human primates receiving intracranial PBM provide further evidence that PBM has an acceptable safety profile (Darlot et al., 2016; Moro et al., 2017).

While transcranial PBM is an effective approach for mitigating PD‐related features in small animal models, the substantial attenuation of light energy by the thick human scalp and skull presents a major barrier to the clinical use of transcranial PBM for the purpose of neuroprotection. Although some recent small clinical studies have reported the promising observation that transcranial PBM improves some signs and symptoms in people with PD (Bullock‐Saxton et al., 2021; Hamilton et al., 2019; Liebert et al., 2021; Santos et al., 2019), the rapidity of the phenotypic responses in these trial participants suggests that these effects arise from acute improvement of neuronal function. Still untested is whether the common progression of the condition has been stopped or stabilized. Measurements in post‐mortem human tissue indicate that only 1%–3% of emitted light penetrates the skull (Hart & Fitzgerald, 2016), while measurements in living rodents indicate that (for 670 nm light) ~65% of light energy is lost for every millimetre of brain tissue traversed (Moro et al., 2014), making the intensity of light reaching the midbrain vanishingly low. One alternative approach is to deliver light intracranially, which has been shown to successfully mitigate dopaminergic cell loss in MPTP‐treated mice (Moro et al., 2014), mitigate dopaminergic cell loss and apomorphine‐induced rotational behaviour in a 6‐hydroxydopamine rat model of PD (Reinhart et al., 2016), and mitigate dopaminergic cell and axon terminal loss and improve clinical scores in MPTP‐treated non‐human primates (Darlot et al., 2016). Intracranial PBM thus shows considerable promise but requires invasive neurosurgery and expensive implantable devices.

Exploring alternative ways of delivering PBM, we have previously reported the phenomenon of ‘remote PBM’, whereby the application of PBM treatment to peripheral tissues also provides protection of the brain (Gordon et al., 2019; Kim et al., 2017). Using a per‐conditioning intervention in an MPTP mouse model of PD, we found that applying PBM to the body of the animal, while shielding the head from transcranial irradiation, mitigates the loss of functional dopaminergic neurons in the substantia nigra pars compacta (SNc) (Johnstone et al., 2014; Stone et al., 2013). This observation was validated in subsequent studies, where remote PBM was delivered as a pre‐conditioning intervention (Ganeshan et al., 2019) or in a different mouse strain (Kim et al., 2018).

The discovery of a systemic protective effect of PBM has the potential to overcome major barriers associated with clinical translation of PBM therapy for PD, raising the question of whether there is an optimal remote PBM target site for inducing neuroprotection of the brain. As a step towards building this understanding, we herein report a comparison of the abdomen and legs as targets for remote PBM, first through *n* = 1 pilot studies in an MPTP non‐human primate model of PD, followed by validation studies in a larger cohort of an MPTP mouse model of PD.

## 2 MATERIALS AND METHODS

### 2.1 Macaque experiments

#### 2.1.1 Ethics statement

All experiments were approved by the Animal Ethics Committee COMETH (Grenoble) and the French Ministry for Research (protocol number 2015062911349260) and were performed in accordance with the European Communities Council Directive of 1986 (86/609/EEC) for the care of laboratory animals. In accordance with animal welfare guidelines, animals were maintained in squeeze‐back cages under controlled conditions of temperature and light (12‐h light/dark cycle), were fed regularly on a diet of fruit, vegetables and pellets and had ad libitum access to water.

#### 2.1.2 MPTP injection and PBM treatment

Adult male macaques (*n* = 3), aged 4–5 years and weighing 5–7 kg, were restrained in squeeze‐back home cages and injected intramuscularly with MPTP (.3 mg/kg/day) over five consecutive days (Days 1–5). Immediately following and 4 h after injection, restrained animals received 670‐nm photobiomodulation (50 mW/cm^2^) from a WARP 10 device (Quantum Devices Inc.), directed at either the head (180 s, 9 J/cm^2^), abdomen (180 s, 9 J/cm^2^) or lower legs (90 s per leg, 4.5 J/cm^2^). The full experimental period extended to Day 21—from Day 6 to Day 21, animals underwent daily clinical evaluation but did not receive MPTP injection nor PBM treatment.

#### 2.1.3 Clinical evaluation

Animals underwent daily clinical evaluation in their home cages during the 21 day experimental period by an investigator blinded to the experimental grouping. Animals were scored (0–3) for 22 clinical signs (e.g., posture, equilibrium, general activity, tremor and bradykinesia) using a modified J. S. Schneider scale (Schneider et al., 2003), to generate a composite score ranging between 0 (*no clinical signs*) and 66 (*severe clinical signs*).

#### 2.1.4 Tissue collection, processing and immunohistochemistry

At the end of the 21‐day experimental period, animals were euthanized by intramuscular injection with 60 mg/kg sodium pentobarbital. Animals were perfused transcardially with a saline clearing solution followed by 4% buffered paraformaldehyde. Brains were removed, post‐fixed overnight then cryoprotected in 30% sucrose in phosphate‐buffered saline (PBS). Serial coronal brain sections (50 μm) were generated using a freezing microtome and collected into PBS in five alternate series (20–25 sections per series). Sections were blocked in 10% normal mouse serum in PBS (1 h), permeabilized in 1% Triton in PBS (1 h) and then incubated in anti‐tyrosine hydroxylase (TH) primary antibody (T8700, Sigma) at 4°C for 48 h, to label dopaminergic cells. Sections were then incubated in biotinylated anti‐rabbit IgG for 4 h and ExtrAvidin‐peroxidase complex for 2 h at room temperature (EXTRA3‐1KT, Sigma). Bound antibody was visualized by incubating sections with 3,3′‐diaminobenzidine tetrahydrochloride solution. Sections from the SNc were additionally subjected to a Nissl stain using cresyl violet, in order to visualize all neurons (including surviving neurons that had lost TH expression). Sections were mounted onto gelatinized slides, dehydrated through increasing concentrations of ethanol, cleared in histolene and coverslipped using DPX mountant. To avoid inter‐experimental variability in the immunohistochemical reaction, all sections were labelled in a single experiment.

#### 2.1.5 Neuron quantification

Quantification of the number of neurons and density of terminals was undertaken by an investigator blinded to sample identity. The number of TH^+^ and Nissl‐stained cells in the SNc was estimated using the optical fractionator method within the Stereo Investigator software package (MBF Bioscience) (Darlot et al., 2016). Briefly, using a counting frame of standardized dimensions (45 × 45 μm) within a grid size of 108 × 81 μm, at least 10 random sites within the defined boundaries of the SNc were sampled per section, and all cells that came into focus within the counting frame were counted. Sections were sampled throughout the volume of the SNc (1:5 series, 20–25 sections per animal).

The density of TH^+^ terminals in the dorsal striatum was quantified in five sections per animal. Brightfield images were captured under standard illumination conditions and imported into ImageJ. Each image was processed in an identical manner, whereby the colour threshold was adjusted to a set level and the mean grey value was measured for each image, as described previously (Darlot et al., 2016).

#### 2.1.6 Data analysis

For comparisons to saline controls and untreated MPTP non‐human primates, we have drawn on data from a previous cohort of animals undergoing an identical experimental regime (Darlot et al., 2016); such instances are made explicit in the text.

### 2.2 Mouse experiments

#### 2.2.1 Animals

All experiments used male C57BL/6 mice, aged approximately 10 weeks at the start of experimentation. Mice were randomly assigned to cages of five and exposed to a 12‐h light/dark cycle, with ad libitum access to food and water.

#### 2.2.2 MPTP injections

Mice were subjected to an acute MPTP regimen, in which they received four intraperitoneal injections of MPTP in saline, with a 2‐h period between each injection. A concentration of 12.5‐mg/kg MPTP was delivered with each injection, for a final dose of 50 mg/kg. Injection volumes ranged between .6 and 1 ml, depending on mouse body weight. A control group of mice were injected with equivalent volumes of saline vehicle. Following injections, mice were observed closely for 24 h to monitor any marked deterioration in health.

#### 2.2.3 Photobiomodulation treatment

To enable PBM to be targeted exclusively at the head, abdomen or legs of a mouse, we designed, engineered and constructed an LED device with a spot size of ~3 cm^2^, as opposed to the 10‐cm^2^ spot size of the WARP 10. The device consisted of a tunable deep red Luxeon® 7 Rebel LED module coupled to a 3D‐printed housing to limit the spot size radius to 1 cm. Various tests were conducted to ensure the device was suitable for use, including ensuring that (1) the interface did not react with the animal's skin, (2) the device can tolerate interaction with mice, (3) the device can be reproducibly manufactured, (4) the electrical componentry can be isolated, (5) the device can be sanitized, (6) the device is stable over multiple days of use, (7) the device has a consistent irradiance and spectral output and (8) the device does not emit excessive heat or noise. These outcomes were met, with measurements confirming that the device emits continuous wave red light at an irradiance of 50 mW/cm^2^ and peak wavelength of 656 nm. Measurements of heat and sound emitted from the device indicate that the increase in device temperature over 5 min of continuous use is 1.4°C, while the emitted sound is 61.8 dB (the volume of normal conversation).

Prior to PBM treatment, an electric groomer with a 20‐mm trimmer attachment (Remington, PG350) was used to shave the fur on the head, hind legs and abdomen, in order to eliminate the potential for light absorption by the black fur. Each PBM treatment session commenced at 9:00 am, with the first session commencing the morning after MPTP/saline injections. Treatments occurred daily over the 21‐day survival period. To minimize any confounding effect of potential circadian differences, the order in which the experimental groups were treated was randomized each day.

Mice were manually restrained and administered one of three PBM treatment protocols, wherein light was exclusively targeted at the shaved region of either the head, abdomen or leg. When irradiating the leg, a light‐opaque shield was used to enable irradiation of the limb while avoiding unintended irradiation of the abdomen or other body parts. For mice treated to the abdomen and head, the irradiation site was consistent throughout the 180‐s treatment period (total dose of 9 J/cm^2^), whereas for mice treated to the leg, each limb was separately irradiated for 90 s for a dose of 4.5 J/cm^2^. Sham treatments involved manually restraining the mice for 180 s, as per the PBM groups, with the devices held to the mice but not emitting any light.

#### 2.2.4 Vertical pole test

The vertical pole test evaluates the ability of a mouse to grasp, manoeuvre and descend a pole and is widely used to assess basal ganglia‐associated movement disorders in rodents. A pole measuring 1 cm in diameter and 55 cm in length was erected within a home cage to encourage descent into the familiar environment. To train mice in the task, mice were placed facing downwards on the vertical pole for a single trial to encourage descent into the home cage. For formal testing, mice were handled by the operator for 30 s to become habituated to manual handling. Trials were initiated by placing the mouse at the top of the pole, facing upwards. Once it was adequately supporting its weight, a cap was placed above the mouse to ensure it did not ascend the pole. The mouse would then turn and descend the pole, with each trial filmed with a digital camera (Canon EOS M3 Mirrorless DSLR, Tokyo, Japan). A total of five trials were completed for each mouse, with approximately 3 min between each trial. Between each cage, the investigator's gloves and the vertical pole were cleaned with 70% ethanol to prevent olfactory cues which may alter behaviour.

Videos were played in VideoLAN Client (VLC) media player with VLC extension Time v3.2, which provides an accurate running time on the screen of a playing video. Two measures of mouse performance were quantified: the time to turn 180° with both paws facing downwards (‘time to turn’) and the total time to descend the entire 50‐cm testing region of the pole (‘time to descend’). The fastest time over the five trials was recorded for each mouse. Mice were tested at baseline (i.e., before MPTP/saline injected) and at 9 days post‐injection. The performance of each mouse at Day 9 post‐injection was normalized to their corresponding baseline performance.

#### 2.2.5 Tissue collection and processing

Mice were euthanized by intraperitoneal injection of sodium pentobarbital (60 mg/kg) and perfused transcardially with PBS. Brains were harvested and immersion fixed in 10% formalin in PBS at 4°C for 24 h. Brains were bisected along the midline and a hemisphere sliced in the coronal plane at the optic chiasm and the superior colliculus (approximately 0 to −5 mm Bregma) to produce a tissue block containing the nigrostriatal pathway. Tissue blocks were then cryoprotected in 30% sucrose in PBS, embedded in Tissue‐Tek OCT compound and frozen in methyl‐2‐butane cooled in liquid nitrogen, and sectioned on a Leica CM 3050 S Research Cryostat (Wetzlar, Germany). For the SNc, tissue was sectioned at 30‐μm thickness into three alternate series (14–18 sections per series); for the caudate–putamen complex (CPu), tissue was sectioned at 20 μm thickness into six alternate series (8–10 sections per series). Sections were collected on poly‐l‐lysine and gelatin coated slides and stored in slide boxes at −80°C until required for immunohistochemistry.

#### 2.2.6 Immunohistochemistry

Slides were dried at 40°C for 1 h, immersed in 70% ethanol for 5 min to remove residual OCT, and rinsed briefly in PBS followed by 1% Triton (Sigma‐Aldrich) in PBS. Sections were incubated in 1.5% normal goat serum for 1 h on a platform rocker, in order to block non‐specific antibody binding, followed by permeabilization in 1% Triton in PBS for 1 h. After rinsing in PBS, sections were incubated in .2% rabbit anti‐tyrosine hydroxylase polyclonal primary antibody (T8700; Sigma‐Aldrich) in PBS at 4°C for 48 h. After rinsing in PBS, sections were incubated in .5% biotinylated anti‐rabbit secondary antibody in 1.5% normal goat serum (VECTASTAIN® Elite ABC‐HRP Peroxidase Kit) on a platform rocker at room temperature for 4 h. After rinsing in PBS, sections were incubated in streptavidin‐HRP solution (VECTASTAIN® Elite ABC‐HRP Peroxidase Kit) on a platform rocker at room temperature for 2 h. After rinsing with PBS, sections were incubated with .2% (w/v) 3,3′‐diaminobenzidine tetrahydrochloride (DAB) to visualize antibody labelling. Sections from the SNc were then counterstained with cresyl violet (i.e., Nissl stain). Sections were dehydrated by immersing slides in ascending concentrations of ethanol, followed by clearing in two exchanges of histolene and coverslipping with DPX mountant. To avoid inter‐experimental variability in the immunohistochemical reaction, all sections were labelled in a single experiment.

#### 2.2.7 Neuron quantification

The numbers of TH^+^ cells and Nissl‐stained neurons in the SNc were estimated using the optical fractionator function provided by the Stereo Investigator software. Prior to cell counting, the identity of slides was concealed to ensure adequate blinding and mitigate potential operator bias. A counting frame of standardized dimensions (45 × 45 μm) within a grid size of 108 × 81 μm resulted in approximately 20 sites for counting per section. Six sections were counted for each animal: two caudal (~Bregma −3.80 mm), two rostral (~Bregma −3.08 mm) and two within these boundaries (~Bregma −3.40 mm). Only cells that were both in focus and strongly labelled were counted. Only Nissl‐stained cells with a size and shape similar to TH‐labelled neurons were counted, thereby omitting smaller (likely non‐neuronal) cells from the counts.

To assess the density of TH^+^ terminals in the CPu, labelled sections (between ~Bregma .74 and −.10) were scanned using an Axio Scan.Z1 Slide Scanner (Zeiss, Germany) and subjected to densitometry analysis using the Fiji imaging processing package. Images were first converted into an 8‐bit greyscale image, then the colour scale was inverted so that stronger labelling corresponded to a higher grey value. A sampling box was drawn in the dorsal CPu region (approximately 1 × 1 cm in size) and mean grey values within this region of interest were captured, while the level of non‐specific background staining was captured by sampling a region of the adjacent neocortex. An adjusted mean grey value was calculated by subtracting the mean grey value of background from the mean grey value of the region of interest (i.e., the CPu). The adjusted mean grey value was measured in the five most rostral sections collected from each animal and the mean calculated.

#### 2.2.8 Data analysis

Statistical analysis and data visualization were conducted using GraphPad Prism 9. One‐way ANOVA was used to compare group means; in cases where *p* < .05, post hoc pairwise comparisons were made using Tukey's multiple comparison test.

## 3 RESULTS

### 3.1 Remote PBM mitigates clinical signs and neuropathology in MPTP‐injected non‐human primates

Adult male macaques (*n* = 3) were injected with MPTP and received PBM directed at either the head, lower legs (tibiae) or abdomen. Clinical scores and neuropathological findings were compared across these three non‐human primates and also to control data from a well‐powered study (*n* = 16) published previously by our team (Darlot et al., 2016). This previous study used non‐human primates of the same species, age and sex as the present study, and experiments were conducted in the same facility and using the same protocols.

**FIGURE 1:**
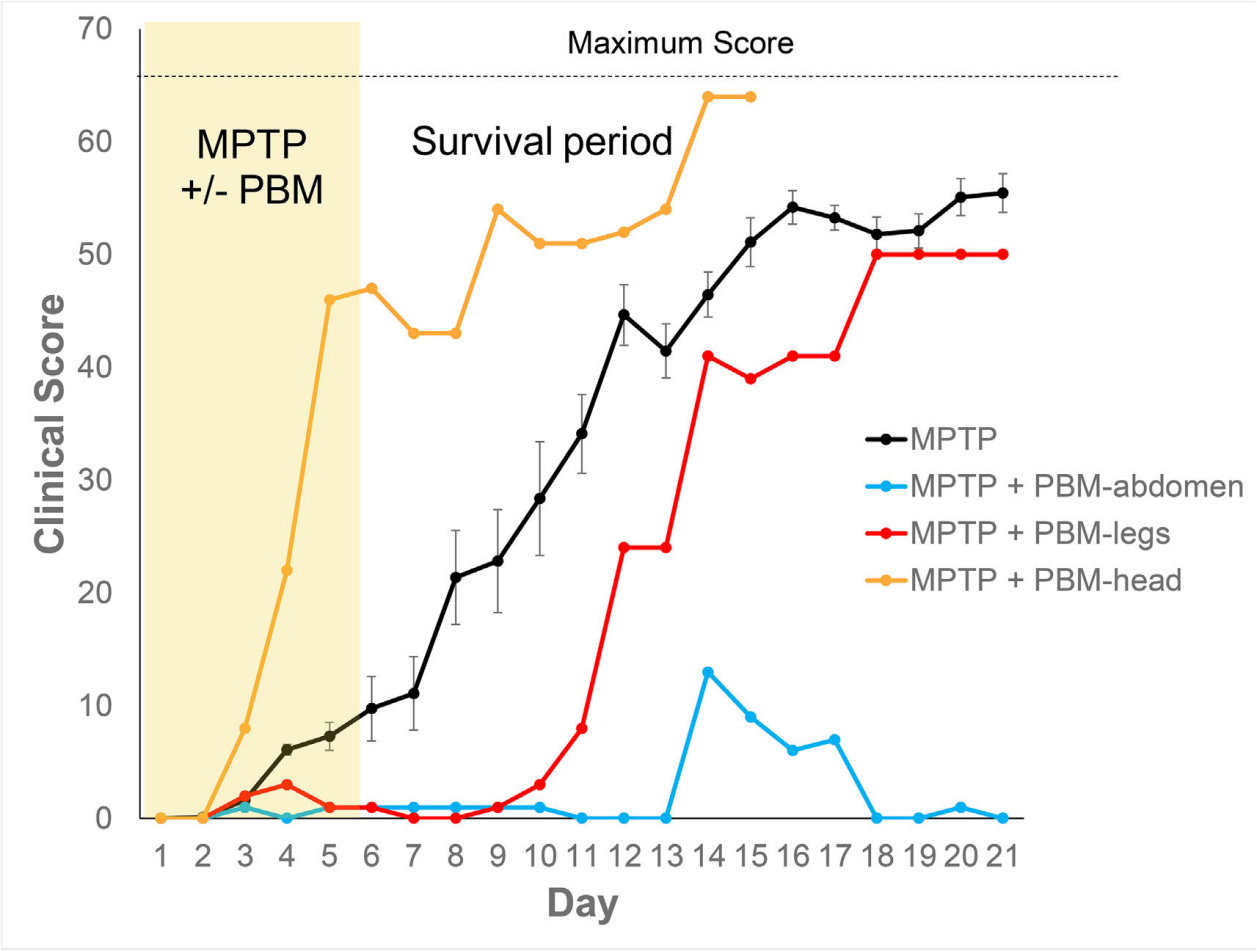
Clinical scores of MPTP‐injected non‐human primates with and without photobiomodulation (PBM) treatment. Scoring of clinical signs utilized a modified J. S. Schneider scale (22 parameters, maximum score = 66). Non‐human primates were scored daily over the 21‐day experimental period, prior to any scheduled injections or PBM treatments. The MPTP‐injected animal that received PBM to the head was terminated at Day 15. Error bars in the MPTP group (*n* = 11) indicate SEM.

Clinical scores are shown in Figure 1. The non‐human primate receiving PBM to the head (transcranial) exhibited no benefit of treatment; it showed severe parkinsonian signs by Day 5 and required termination by Day 15, 1 week before the scheduled end of the experimental period. In contrast, both non‐human primates receiving remote PBM were visibly healthier. For the non‐human primate receiving PBM to the lower legs, the onset of clinical signs was delayed by 7 days, although by the end of the experimental period, the animal had developed a phenotype of similar severity to untreated MPTP non‐human primates. In contrast, the non‐human primate receiving PBM to the abdomen exhibited only minor clinical signs and by the end of the experimental period exhibited no behavioural manifestations of MPTP insult.

**FIGURE 2:**
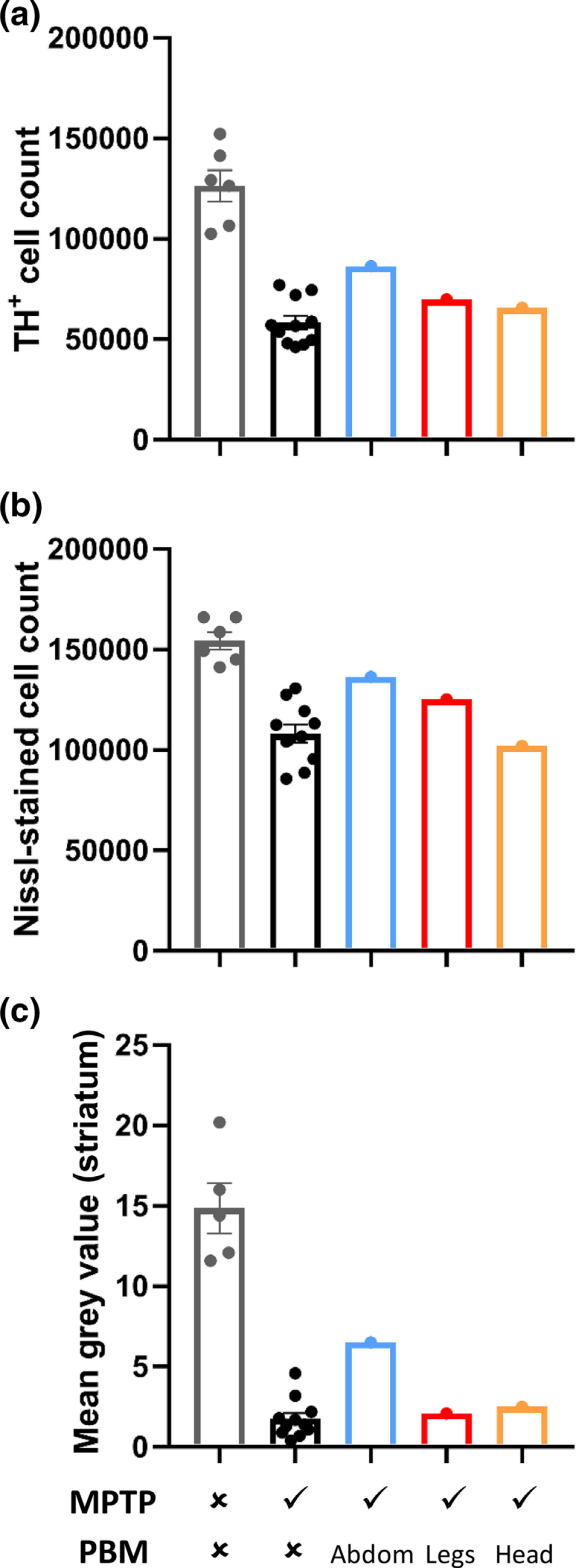
Immunohistochemical quantification of nigrostriatal pathology in non‐human primates. (a) Stereological counts of tyrosine hydroxylase‐positive (TH^+^) cells in the substantia nigra pars compacta (SNc), as a measure of functional dopaminergic neurons. (b) Stereological counts of Nissl‐stained neurons in the SNc, as a measure of total surviving neurons. (c) Densitometry analysis of TH labelling in the striatum, as a measure of dopaminergic terminals. Error bars in the control and MPTP groups indicate SEM.

**FIGURE 3:**
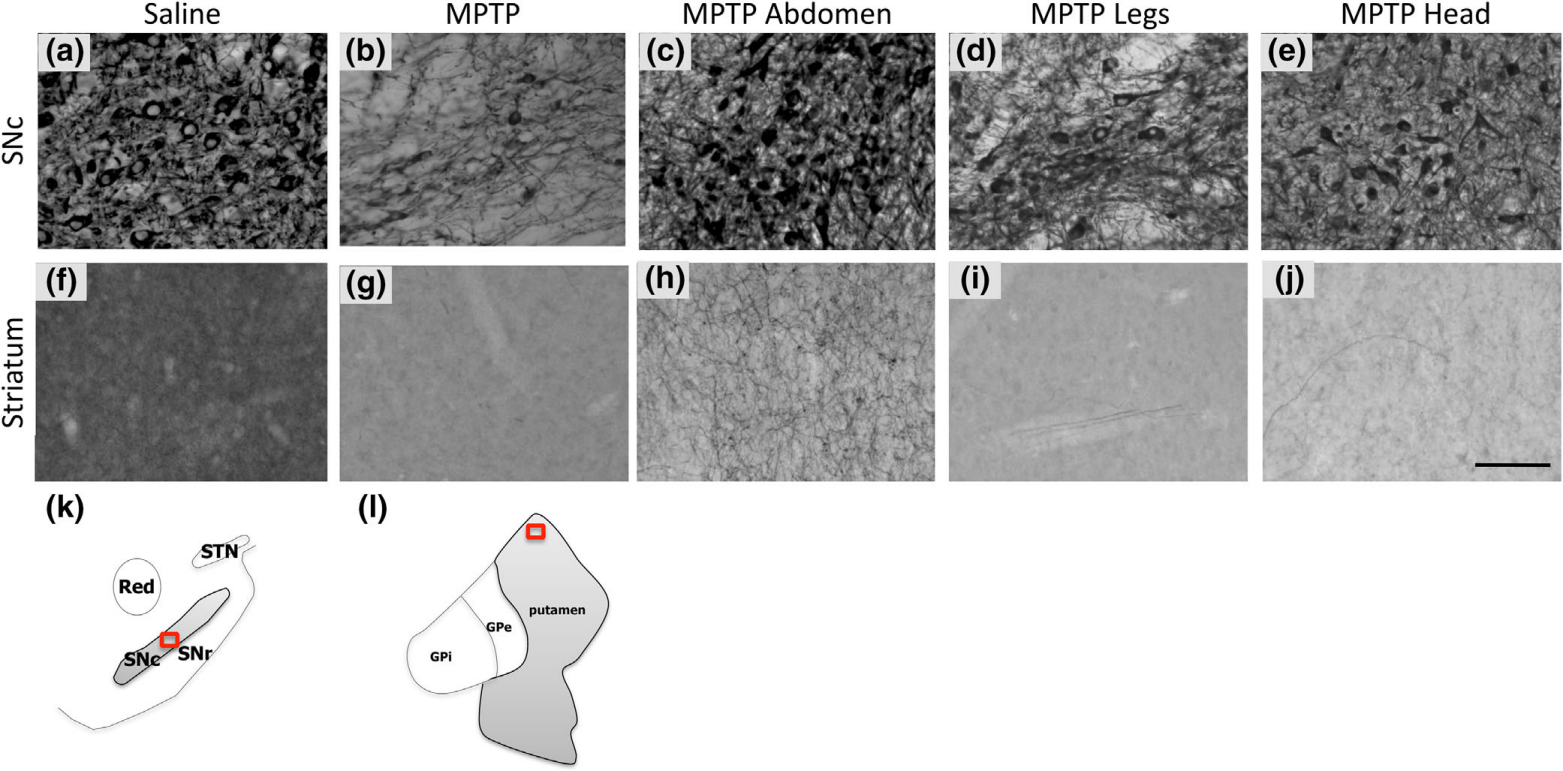
Representative photomicrographs of TH immunohistochemical labelling in the SNc and striatum of non‐human primates. (a–e) TH‐labelled sections from SNc. (f–j) TH‐labelled sections from striatum. (k) For the SNc, photomicrographs were taken from a central region corresponding to plates 69 and 70 of the monkey brain atlas (Paxinos et al., 1999); (l) for the striatum, photomicrographs were taken from a dorsal region corresponding to plates 63 and 64. Scale bar = 100 μm. GPe, globus pallidus externus; GPi, globus pallidus internus; SNr, substantia nigra pars reticulata; STN, subthalamic nucleus.

Dopaminergic cells in the nigrostriatal pathway were visualized by immunohistochemical labelling of tyrosine hydroxylase (TH), the rate‐limiting enzyme of catecholamine biosynthesis. Analysis of the midbrain corroborated the behavioural data (Figures 2a,b and 3). The non‐human primate treated with remote PBM to the abdomen showed ~50% more TH^+^ cells (i.e., dopaminergic cells) in the SNc than a cohort of untreated MPTP non‐human primates, and over 25% more Nissl stained cells (i.e., neurons). Remote PBM to the lower legs produced a more modest rescue of dopaminergic cells (20%) and total neurons (15%). The MPTP non‐human primate treated with transcranial PBM showed similar cell counts to untreated MPTP non‐human primates.

We also assessed the density of TH^+^ terminals in the striatum (Figures 2c and 3). Consistent with the midbrain cell counts, the non‐human primate treated with remote PBM to the abdomen showed a 3.7‐fold greater density of TH labelling in the striatum than the cohort of untreated MPTP non‐human primates. There was no appreciable rescue of TH^+^ terminals in the non‐human primates treated to either the lower legs or the head.

### 3.2 Remote PBM mitigates nigrostriatal degeneration in MPTP‐injected mice

**FIGURE 4:**
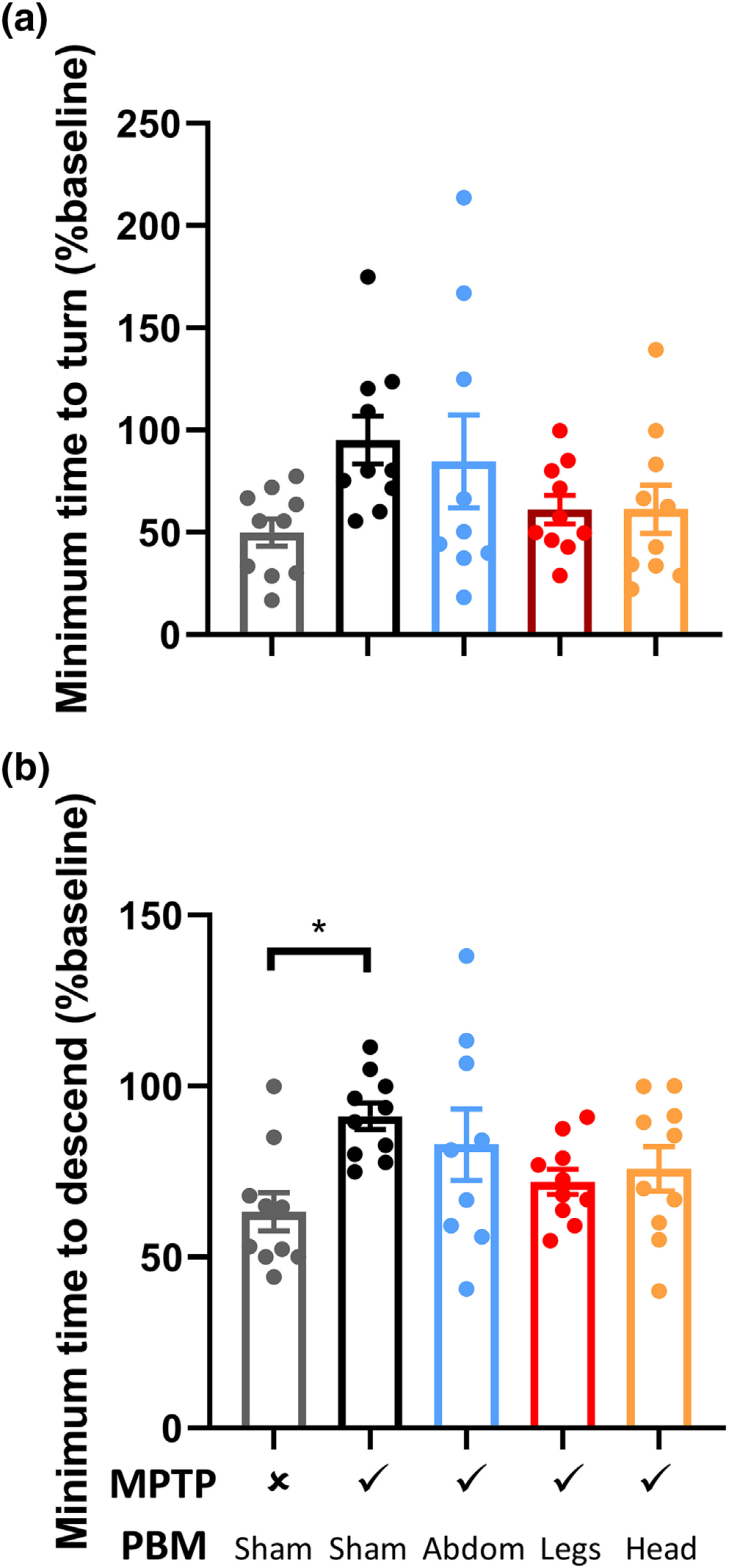
Vertical pole testing of motor coordination in mice. Vertical pole testing was conducted on Day 9 post‐injection and normalized to a pre‐injection baseline for each mouse. (a) Minimum time to turn 180° on the vertical pole, from five trials. (b) Minimum total time to descend the vertical pole, from five trials. *p < .05.

To validate the findings from the pilot study of non‐human primates, we assessed the neuroprotective efficacy of PBM targeted at the abdomen or legs in a large cohort of MPTP‐injected mice. To enable PBM to be targeted exclusively to specific regions, we engineered, constructed and validated an LED device with an appropriate spot size of ~3 cm^2^ (irradiance = 50 mW/cm^2^, wavelength = 656 nm) and utilized this device for PBM treatments. Unlike non‐human primates, mice do not manifest obvious and sustained behavioural deficits as a result of MPTP exposure (Jackson‐Lewis & Przedborski, 2007; Meredith & Rademacher, 2011). To investigate more subtle behavioural effects, motor coordination was tested on Day 9 post‐injection using the vertical pole test, and outcome measures were normalized to baseline (i.e., pre‐injection) performance (Figure 4). While there were no significant intergroup differences when considering the ‘time to turn’ (one‐way ANOVA *F*[4, 44] = 2.17, *p* = .09), there were significant intergroup differences in the ‘time to descend’ the pole (one‐way ANOVA *F*[4, 44] = 2.9, *p* = .03). Post‐hoc testing revealed that sham‐treated MPTP mice were slower to descend the pole than saline‐injected controls (*p* = .01), whereas PBM‐treated MPTP mice were not significantly different from saline‐injected controls (*p* > .05). In view of the limited behavioural phenotype of the MPTP mice, outcome measures focused on histology of the nigrostriatal pathway.

**FIGURE 5:**
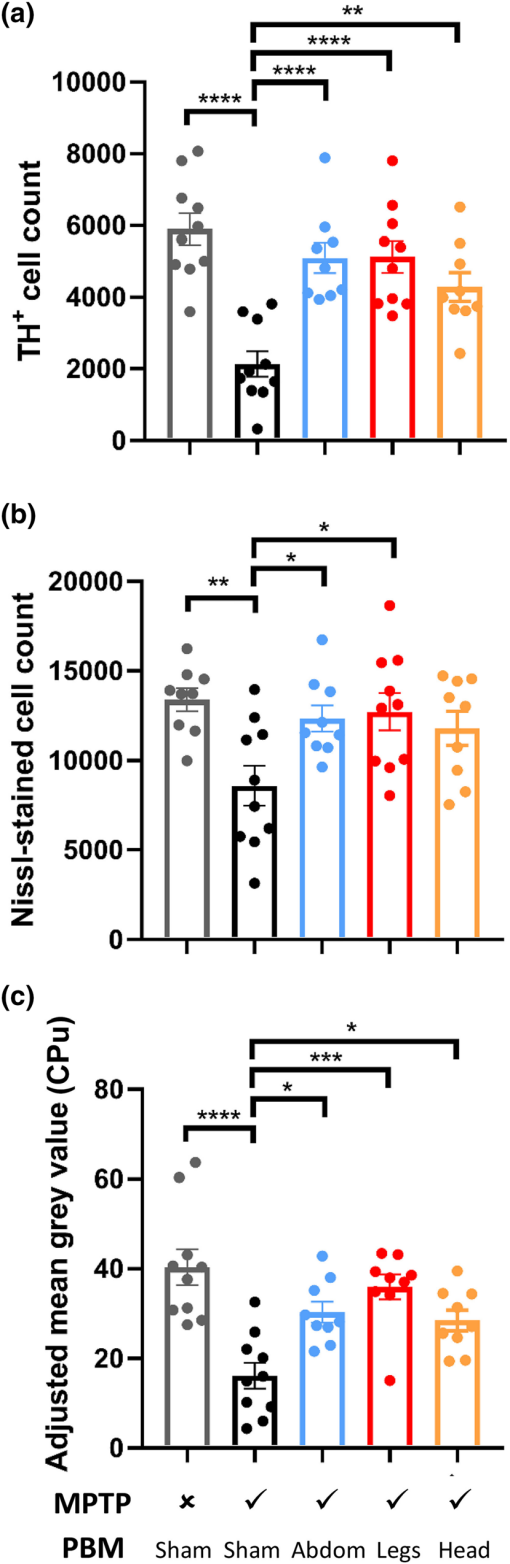
*Immunohistochemical quantification of nigrostriatal pathology in mice. (a) Stereological counts of tyrosine hydroxylase‐positive (TH^+^) cells in the substantia nigra pars compacta (SNc). (b) Stereological counts of Nissl‐stained neurons in the SNc. (c) Densitometry analysis of TH labelling in the caudate–putamen complex. Error bars indicate SEM. *p < .05, **p < .01, ***p < .001, ****p < .0001.

**FIGURE 6:**
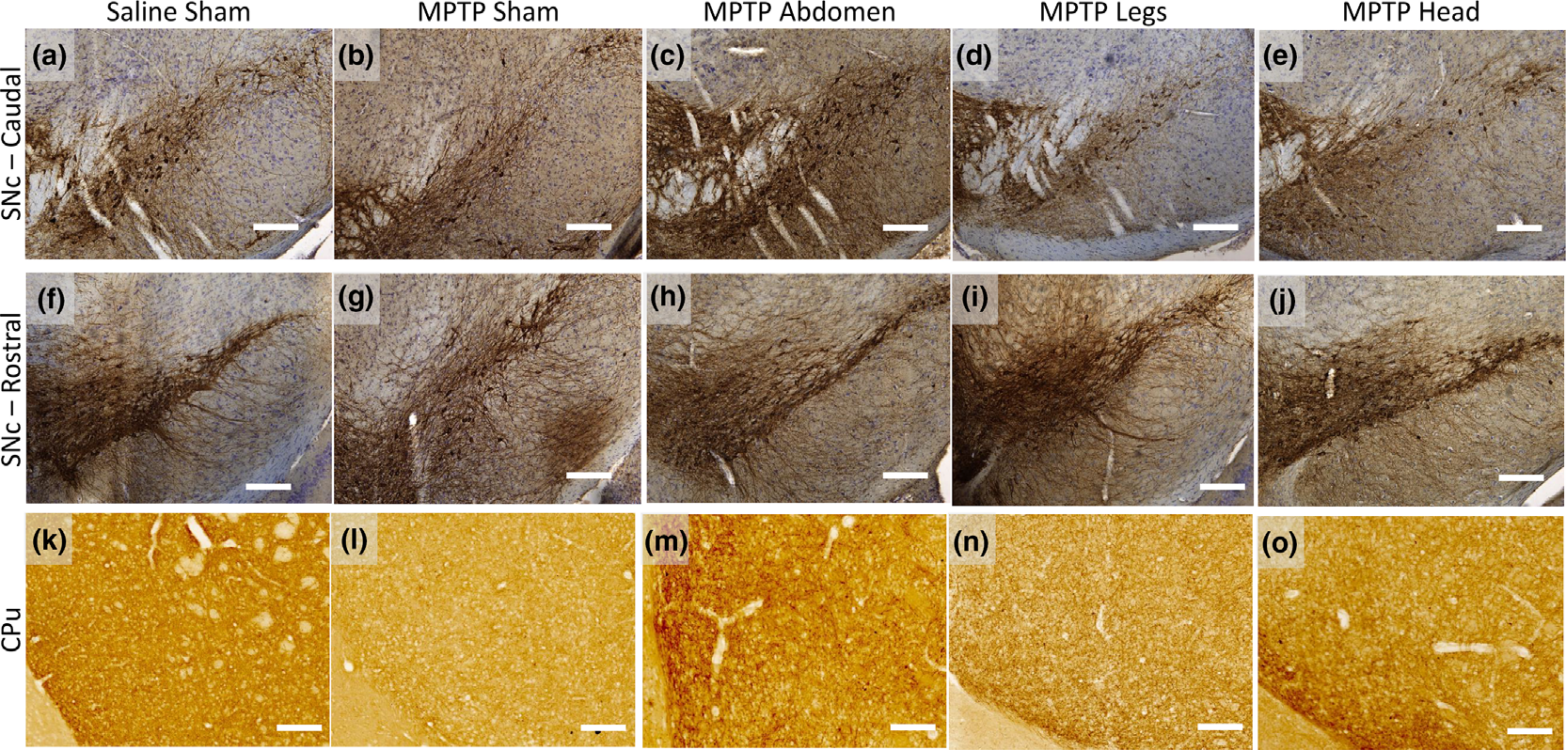
Representative photomicrographs of TH immunohistochemical labelling in the SNc and CPu of mice. (a–e) TH‐labelled sections from caudal SNc (Bregma −3.80 mm), with Nissl counterstain. (f–j) TH‐labelled sections from rostral SNc (Bregma 2.92 mm), with Nissl counterstain. (k–o) TH‐labelled sections from rostral CPu (Bregma .14 mm). All images were taken at ×10 magnification, scale bars = 100 μm.

The number of TH^+^ cells in the SNc was quantified by stereology to provide a measure of the number of dopaminergic neurons (Figures 5a and 6). One‐way ANOVA revealed significant variation in TH^+^ cell counts across the experimental groups (*F*[4, 43] = 12.48, *p* < .0001). Post‐hoc testing revealed significantly fewer TH^+^ cells in sham‐treated MPTP mice than in saline‐injected controls (64% reduction, *p* < .0001). Relative to sham‐treated MPTP mice, there was a significant rescue of TH^+^ cells in MPTP‐injected mice treated with PBM to the head (57% increase, *p* < .01), abdomen (79% increase, *p* < .0001) and legs (79% increase, *p* < .0001).

Nissl‐stained neurons were counted to estimate the total number of neurons in the same sections (Figure 5b). The findings mirrored those above for TH^+^ cells, although differences were slightly less pronounced. One‐way ANOVA indicated significant variation in Nissl‐stained neuron counts across the experimental groups (*F*[4, 42] = 4.185, *p* < .01). Post‐hoc testing revealed significantly fewer Nissl‐stained cells in sham‐treated MPTP mice than in saline‐injected controls (36% reduction, *p* < .01). Relative to sham‐treated MPTP mice, there was a significant rescue of Nissl‐stained cells in MPTP‐injected mice treated with PBM to the abdomen (78% increase, *p* < .05) and legs (86% increase, *p* < .05) but not the head (*p* > .05).

To complement the analysis of the SNc, we also assessed the density of TH^+^ terminals in the CPu (Figures 5c and 6). One‐way ANOVA revealed significant variation in the density of TH labelling, as assessed by adjusted mean grey value, across the experimental groups (*F*[4, 42] = 9.913, *p* < .0001). Post‐hoc testing revealed a significantly lower density of TH^+^ terminals in sham‐treated MPTP mice than in saline‐injected control mice (60% reduction, *p* < .0001). Relative to sham‐treated MPTP mice, there was a significant rescue of TH^+^ terminals in MPTP‐injected mice treated with PBM to the head (51% increase, *p* < .05), abdomen (58% increase, *p* < .05) and legs (82% increase, *p* < .001).

## 4 DISCUSSION

This study confirms that remote PBM provides neuroprotection against MPTP insult and identifies the abdomen and lower legs as viable PBM targets for remote rescue of the nigrostriatal pathway. Importantly, the degree of neuroprotection provided by remote PBM was at least as, if not more, effective than transcranial PBM.

In mice, remote PBM targeted at either the abdomen or legs mitigated MPTP‐induced loss of TH^+^ cell bodies in the SNc and terminals in the CPu, and also Nissl‐stained cells in the SNc. The complementary approaches of TH immunohistochemistry and Nissl staining allowed us to distinguish between what we have previously described as true neuroprotection and functional neuroprotection (Darlot et al., 2016), that is, does remote PBM protect neurons from degeneration or simply restore a dopaminergic phenotype in neurons that are damaged but surviving? The findings presented here suggest that remote PBM induces both true and functional neuroprotection.

It is important to acknowledge that the pilot non‐human primate data presented here are underpowered and should not be used as the basis for any firm conclusions. In addition, despite being considered the gold standard animal model of PD, non‐human primates have been reported to show some variability in the phenotypic manifestations of MPTP insult (Potts et al., 2014). Nonetheless, we believe that they add value to the more rigorous mouse model data; indeed, the non‐human primate observations served as the motivation for undertaking the mouse validation study. First, all evidence for neuroprotection with remote PBM to date has been derived from rodent models; these pilot data from non‐human primates, while underpowered, provide early clues that a similar phenomenon might also occur in primates, with obvious implications for clinical translation. Second, the non‐human primate observations provide preliminary insights into the effect of remote PBM on the clinical signs of PD, which are readily manifested by MPTP‐injected monkeys but not by MPTP‐injected mice. Notwithstanding the obvious limitations of the non‐human primate data, the observation of a potentially profound effect of remote PBM (particularly abdomen‐targeted PBM) on MPTP‐induced clinical signs should serve as motivation for further testing. While the PBM‐induced mitigation of nigrostriatal damage in MPTP‐injected non‐human primates was far less pronounced than that in MPTP‐injected mice, it is important to note that monkeys were only treated with PBM for the 5 day duration of the MPTP injection protocol. In contrast, mice were subjected to a post‐conditioning protocol, where PBM treatments commenced the day after the completion of MPTP injections and were continued throughout the survival period. It will be valuable to determine whether extended periods of remote PBM treatment, which align more closely with a clinical therapeutic strategy, confer stronger symptomatic and neuroprotective effects in large primate species (whether non‐human primates or humans).

An additional limitation relates to the exclusive use of male animals in the present study. This is common practice when utilizing MPTP mouse models, as it is well established that female mice are more susceptible to cardiovascular side effects of MPTP intoxication, and as a result have a higher death rate (Jackson‐Lewis & Przedborski, 2007). Furthermore, a recent comparison of MPTP‐injected male and female mice demonstrated that, unlike male mice, female mice manifest little or no PD‐related neuropathology as a result of MPTP intoxication (Isenbrandt et al., 2021). In terms of non‐human primates, the choice of male animals was pragmatic, as it enabled direct comparison with a previous, well‐powered study conducted by our team (Darlot et al., 2016), which exclusively used male macaques. It will be important for future studies, using different models, to confirm that the neuroprotective effects of remote PBM are robust across both sexes.

While MPTP induces obvious parkinsonism in non‐human primates, a limitation of MPTP mouse models is the lack of a consistent and overt motor phenotype. In the present study, we utilized the vertical pole test, which has been previously suggested to detect bradykinesia in MPTP‐injected mice (Ogawa et al., 1985).While sham‐treated MPTP mice took significantly longer to descend the pole than saline‐injected controls, there was no significant difference between sham‐treated and PBM‐treated MPTP mice. The high degree of intra‐group variability suggests that the study may have been underpowered to detect any effect of PBM on this particular outcome measure, hence our focus on the neuropathological outcomes.

The intriguing observation of remote PBM‐induced neuroprotection raises obvious questions as to the mechanism. The decision to target PBM at the lower legs was based on previous work by Tuby et al. (2011) suggesting that tibial bone marrow‐derived stem cells, possibly mesenchymal stem cells, are responsible for mediating the body‐wide protective effects of PBM. Adipose tissue is also a rich source of mesenchymal stem cells (Miana & Gonzalez, 2018), raising the possibility that adipose tissue‐derived stem cells mediate the observed neuroprotective effects of abdomen‐targeted PBM. As noted previously, mesenchymal stem cells are a viable candidate for mediating neuroprotection, as they respond to PBM treatment, can transmigrate across the blood–brain barrier, home specifically to areas of pathology and damage and release trophic factors that can facilitate the repair of damaged cells (Johnstone et al., 2015).

In addition to circulating cells, circulating molecules such as cytokines, chemokines and/or mitokines might also be involved in mediating remote PBM‐induced neuroprotection, as reviewed previously (Gordon et al., 2019). In addition, the beneficial effects of abdomen‐targeted remote PBM might be driven by circulating gut‐derived neuropeptides that have neuroprotective actions, such as ghrelin (Bayliss et al., 2016). Alternatively, abdomen‐targeted remote PBM might modulate the dopamine‐rich enteric nervous system, and in turn the gut–brain axis, which many investigators now propose is important in PD pathogenesis (Klingelhoefer & Reichmann, 2015). Finally, there is mounting evidence that PBM of the abdomen positively influences the gut microbiome (Bicknell et al., 2019, 2022, 2022; Liebert et al., 2019), which shows dysbiosis in people with PD (Romano et al., 2021) and has been proposed as a potential contributor to PD pathogenesis (Elfil et al., 2020).

In conclusion, the findings of this study add to the mounting evidence that PBM has indirect (likely systemic) effects and that remote PBM is a promising neuroprotective intervention. This will hopefully motivate a refocusing of PBM clinical trials, which predominantly have been designed to investigate transcranial PBM. Indeed, there has already been a detectable shift in approach, with at least one trial designed to evaluate remote PBM showing positive effects on clinical signs (Liebert et al., 2022). More pre‐clinical research will follow to determine the robustness of the neuroprotective effect (e.g., in non‐MPTP PD models), to optimize PBM parameters (e.g., wavelength, irradiance, dose and treatment frequency), to personalize treatment strategies to account for intersubject variability and to unravel the mechanisms underpinning remote PBM‐induced neuroprotection.

## AUTHOR CONTRIBUTIONS

Luke Gordon contributed in the data curation, formal analysis, investigation, methodology, resources, software, validation, visualization and writing the original draft preparation. Kristy Martin helped in the investigation and project administration. Napoleon Torres contributed in the study methodology and resources. Alim‐Louis Benabid was responsible for funding acquisition, methodology, project administration, resources and supervision. John Mitrofanis helped in the investigation, methodology, resources, supervision and writing review and editing. Jonathan Stone contributed to the study conceptualization, supervision and writing review and editing. Cecile Moro helped in the study conceptualization, data curation, investigation, methodology, project administration, resources and writing review and editing. Daniel Johnstone contributed in the study conceptualization, data curation, formal analysis, funding acquisition, investigation, project administration, resources, supervision, visualization, writing the original draft preparation and writing review and editing.

## CONFLICT OF INTEREST STATEMENT

D. M. J. is a founding shareholder in SYMBYX PTY LTD and J.S. is a Director of CSCM Pty Ltd. These companies had no role in the design, conduct or analysis of the study.

## ETHICS STATEMENT

All mouse experiments were approved by the University of Sydney Animal Ethics Committee (protocol number 2017/1128).All non‐human primate experiments were approved by the Animal Ethics Committee COMETH (Grenoble) and the French Ministry for Research (protocol number 2015062911349260) and were performed in accordance with the European Communities Council Directive of 1986 (86/609/EEC) for the care of laboratory animals.

### PEER REVIEW

The peer review history for this article is available at https://www.webofscience.com/api/gateway/wos/peer-review/10.1111/ejn.15973.

## Data Availability

Data will be made available upon request.

## References

[ejn15973-bib-0001] Bayliss, J. A. , Lemus, M. , Santos, V. V. , Deo, M. , Elsworth, J. D. , & Andrews, Z. B. (2016). Acylated but not des‐acyl ghrelin is neuroprotective in an MPTP mouse model of Parkinson's disease. Journal of Neurochemistry, 137, 460–471. 10.1111/jnc.13576 26872221 PMC4836972

[ejn15973-bib-0002] Bicknell, B. , Laakso, E. L. , Liebert, A. , & Kiat, H. (2022). Modifying the microbiome as a potential mechanism of photobiomodulation: A case report. Photobiomodul Photomed Laser Surg, 40, 88–97. 10.1089/photob.2021.0057 34962422

[ejn15973-bib-0003] Bicknell, B. , Liebert, A. , Johnstone, D. , & Kiat, H. (2019). Photobiomodulation of the microbiome: Implications for metabolic and inflammatory diseases. Lasers in Medical Science, 34, 317–327. 10.1007/s10103-018-2594-6 30074108

[ejn15973-bib-0004] Bicknell, B. , Liebert, A. , McLachlan, C. S. , & Kiat, H. (2022). Microbiome changes in humans with Parkinson's disease after photobiomodulation therapy: A retrospective study. Journal of Personalized Medicine, 12, 49. 10.3390/jpm12010049 35055364 PMC8778696

[ejn15973-bib-0005] Blivet, G. , Relano‐Gines, A. , Wachtel, M. , & Touchon, J. (2022). A randomized, double‐blind, and sham‐controlled trial of an innovative brain–gut photobiomodulation therapy: Safety and patient compliance. Journal of Alzheimer's Disease, 90, 811–822. 10.3233/JAD-220467 36189591

[ejn15973-bib-0006] Bullock‐Saxton, J. , Lehn, A. , & Laakso, E. L. (2021). Exploring the effect of combined transcranial and intra‐oral photobiomodulation therapy over a four‐week period on physical and cognitive outcome measures for people with Parkinson's disease: A randomized double‐blind placebo‐controlled pilot study. Journal of Alzheimer's Disease, 83, 1499–1512. 10.3233/JAD-210170 34092640

[ejn15973-bib-0007] Cassano, P. , Norton, R. , Caldieraro, M. A. , Vahedifard, F. , Vizcaino, F. , McEachern, K. M. , & Iosifescu, D. (2022). Tolerability and safety of transcranial photobiomodulation for mood and anxiety disorders. Photonics, 9, 507. 10.3390/photonics9080507

[ejn15973-bib-0008] Darlot, F. , Moro, C. , El Massri, N. , Chabrol, C. , Johnstone, D. M. , Reinhart, F. , Agay, D. , Torres, N. , Bekha, D. , Auboiroux, V. , Costecalde, T. , Peoples, C. L. , Anastascio, H. D. , Shaw, V. E. , Stone, J. , Mitrofanis, J. , & Benabid, A. L. (2016). Near‐infrared light is neuroprotective in a monkey model of Parkinson disease. Annals of Neurology, 79, 59–75. 10.1002/ana.24542 26456231

[ejn15973-bib-0009] Eells, J. T. , Henry, M. M. , Summerfelt, P. , Wong‐Riley, M. T. , Buchmann, E. V. , Kane, M. , Whelan, N. T. , & Whelan, H. T. (2003). Therapeutic photobiomodulation for methanol‐induced retinal toxicity. Proceedings of the National Academy of Sciences of the United States of America, 100, 3439–3444. 10.1073/pnas.0534746100 12626762 PMC152311

[ejn15973-bib-0010] Elfil, M. , Kamel, S. , Kandil, M. , Koo, B. B. , & Schaefer, S. M. (2020). Implications of the gut microbiome in Parkinson's disease. Movement Disorders, 35, 921–933. 10.1002/mds.28004 32092186

[ejn15973-bib-0011] Ganeshan, V. , Skladnev, N. V. , Kim, J. Y. , Mitrofanis, J. , Stone, J. , & Johnstone, D. M. (2019). Pre‐conditioning with remote photobiomodulation modulates the brain transcriptome and protects against MPTP insult in mice. Neuroscience, 400, 85–97. 10.1016/j.neuroscience.2018.12.050 30625333

[ejn15973-bib-0012] Gordon, L. , Kim, B. , Petrucco, C. , Kim, J. Y. , Benson, P. , Stone, J. , & Johnstone, D. M. (2019). Remote photobiomodulation as a neuroprotective intervention—harnessing the indirect effects of PBM. In M. R. Hamblin & Y. Y. Huang (Eds.), Photobiomodulation in the brain: Low‐level laser (light) therapy in neurology and neuroscience (pp. 139–154). Academic Press.

[ejn15973-bib-0013] Hamilton, C. L. , El Khoury, H. , Hamilton, D. , Nicklason, F. , & Mitrofanis, J. (2019). ‘Buckets’: Early observations on the use of red and infrared light helmets in Parkinson's disease patients. Photobiomodulation, Photomedicine, and Laser Surgery, 37, 615–622. 10.1089/photob.2019.4663 31536464

[ejn15973-bib-0014] Hart, N. S. , & Fitzgerald, M. (2016). A new perspective on delivery of red‐near‐infrared light therapy for disorders of the brain. Discovery Medicine, 22, 147–156. 27755969

[ejn15973-bib-0015] Isenbrandt, A. , Morissette, M. , Bourque, M. , Lamontagne‐Proulx, J. , Coulombe, K. , Soulet, D. , & Di Paolo, T. (2021). Effect of sex and gonadectomy on brain MPTP toxicity and response to dutasteride treatment in mice. Neuropharmacology, 201, 108784. 10.1016/j.neuropharm.2021.108784 34555366

[ejn15973-bib-0016] Jackson‐Lewis, V. , & Przedborski, S. (2007). Protocol for the MPTP mouse model of Parkinson's disease. Nature Protocols, 2, 141–151. 10.1038/nprot.2006.342 17401348

[ejn15973-bib-0017] Johnstone, D. M. , el Massri, N. , Moro, C. , Spana, S. , Wang, X. S. , Torres, N. , Chabrol, C. , De Jaeger, X. , Reinhart, F. , Purushothuman, S. , Benabid, A. L. , Stone, J. , & Mitrofanis, J. (2014). Indirect application of near infrared light induces neuroprotection in a mouse model of parkinsonism—An abscopal neuroprotective effect. Neuroscience, 274, 93–101. 10.1016/j.neuroscience.2014.05.023 24857852

[ejn15973-bib-0018] Johnstone, D. M. , Hamilton, C. , Gordon, L. C. , Moro, C. , Torres, N. , Nicklason, F. , Stone, J. , Benabid, A. L. , & Mitrofanis, J. (2021). Exploring the use of intracranial and extracranial (remote) photobiomodulation devices in Parkinson's disease: A comparison of direct and indirect systemic stimulations. Journal of Alzheimer's Disease, 83, 1399–1413. 10.3233/JAD-210052 33843683

[ejn15973-bib-0019] Johnstone, D. M. , Mitrofanis, J. , & Stone, J. (2015). Targeting the body to protect the brain: Inducing neuroprotection with remotely‐applied near infrared light. Neural Regeneration Research, 10, 349–351. 10.4103/1673-5374.153673 25878572 PMC4396086

[ejn15973-bib-0020] Kim, B. , Brandli, A. , Mitrofanis, J. , Stone, J. , Purushothuman, S. , & Johnstone, D. M. (2017). Remote tissue conditioning ‐ an emerging approach for inducing body‐wide protection against diseases of ageing. Ageing Research Reviews, 37, 69–78. 10.1016/j.arr.2017.05.005 28552720

[ejn15973-bib-0021] Kim, B. , Mitrofanis, J. , Stone, J. , & Johnstone, D. M. (2018). Remote tissue conditioning is neuroprotective against MPTP insult in mice. IBRO Reports, 4, 14–17. 10.1016/j.ibror.2018.01.001 30135947 PMC6084900

[ejn15973-bib-0022] Klingelhoefer, L. , & Reichmann, H. (2015). Pathogenesis of Parkinson disease—The gut–brain axis and environmental factors. Nature Reviews. Neurology, 11, 625–636. 10.1038/nrneurol.2015.197 26503923

[ejn15973-bib-0023] Liebert, A. , Bicknell, B. , Johnstone, D. M. , Gordon, L. C. , Kiat, H. , & Hamblin, M. R. (2019). ‘Photobiomics’: Can light, including photobiomodulation, alter the microbiome? Photobiomodul Photomed Laser Surg, 37, 681–693. 10.1089/photob.2019.4628 31596658 PMC6859693

[ejn15973-bib-0024] Liebert, A. , Bicknell, B. , Laakso, E. L. , Heller, G. , Jalilitabaei, P. , Tilley, S. , Mitrofanis, J. , & Kiat, H. (2021). Improvements in clinical signs of Parkinson's disease using photobiomodulation: A prospective proof‐of‐concept study. BMC Neurology, 21, 256. 10.1186/s12883-021-02248-y 34215216 PMC8249215

[ejn15973-bib-0025] Liebert, A. , Bicknell, B. , Laakso, E. L. , Jalilitabaei, P. , Tilley, S. , Kiat, H. , & Mitrofanis, J. (2022). Remote Photobiomodulation treatment for the clinical signs of Parkinson's disease: A case series conducted during COVID‐19. Photobiomodul Photomed Laser Surg, 40, 112–122. 10.1089/photob.2021.0056 34919459

[ejn15973-bib-0026] McGee, C. , Liebert, A. , Herkes, G. , Bicknell, B. , Pang, V. , McLachlan, C. S. , & Kiat, H. (2022). Protocol for randomized controlled trial to evaluate the safety and feasibility of a novel helmet to deliver transcranial light emitting diodes photobiomodulation therapy to patients with Parkinson's disease. Frontiers in Neuroscience, 16, 945796. 10.3389/fnins.2022.945796 36061601 PMC9428720

[ejn15973-bib-0027] Meredith, G. E. , & Rademacher, D. J. (2011). MPTP mouse models of Parkinson's disease: An update. Journal of Parkinson's Disease, 1, 19–33. 10.3233/JPD-2011-11023 PMC3530193 23275799

[ejn15973-bib-0028] Miana, V. V. , & Gonzalez, E. A. P. (2018). Adipose tissue stem cells in regenerative medicine. Ecancermedicalscience, 12, 822. 10.3332/ecancer.2018.822 29662535 PMC5880231

[ejn15973-bib-0029] Moro, C. , Massri, N. E. , Torres, N. , Ratel, D. , De Jaeger, X. , Chabrol, C. , Perraut, F. , Bourgerette, A. , Berger, M. , Purushothuman, S. , Johnstone, D. , Stone, J. , Mitrofanis, J. , & Benabid, A. L. (2014). Photobiomodulation inside the brain: A novel method of applying near‐infrared light intracranially and its impact on dopaminergic cell survival in MPTP‐treated mice. Journal of Neurosurgery, 120, 670–683. 10.3171/2013.9.JNS13423 24160475

[ejn15973-bib-0030] Moro, C. , Torres, N. , Arvanitakis, K. , Cullen, K. , Chabrol, C. , Agay, D. , Darlot, F. , Benabid, A. L. , & Mitrofanis, J. (2017). No evidence for toxicity after long‐term photobiomodulation in normal non‐human primates. Experimental Brain Research, 235, 3081–3092. 10.1007/s00221-017-5048-7 28744621

[ejn15973-bib-0031] Moro, C. , Torres, N. , El Massri, N. , Ratel, D. , Johnstone, D. M. , Stone, J. , Mitrofanis, J. , & Benabid, A. L. (2013). Photobiomodulation preserves behaviour and midbrain dopaminergic cells from MPTP toxicity: Evidence from two mouse strains. BMC Neuroscience, 14, 40. 10.1186/1471-2202-14-40 23531041 PMC3616839

[ejn15973-bib-0032] Ogawa, N. , Hirose, Y. , Ohara, S. , Ono, T. , & Watanabe, Y. (1985). A simple quantitative bradykinesia test in MPTP‐treated mice. Research Communications in Chemical Pathology and Pharmacology, 50, 435–441. 3878557

[ejn15973-bib-0033] Oueslati, A. , Lovisa, B. , Perrin, J. , Wagnieres, G. , van den Bergh, H. , Tardy, Y. , & Lashuel, H. A. (2015). Photobiomodulation suppresses alpha‐synuclein‐induced toxicity in an AAV‐based rat genetic model of Parkinson's disease. PLoS ONE, 10, e0140880. 10.1371/journal.pone.0140880 26484876 PMC4617694

[ejn15973-bib-0034] Paxinos, G. , Huang, X. , & Toga, A. W. (1999). The rhesus monkey brain in stereotaxic coordinates. Academic Press.

[ejn15973-bib-0035] Peoples, C. , Spana, S. , Ashkan, K. , Benabid, A. L. , Stone, J. , Baker, G. E. , & Mitrofanis, J. (2012). Photobiomodulation enhances nigral dopaminergic cell survival in a chronic MPTP mouse model of Parkinson's disease. Parkinsonism & Related Disorders, 18, 469–476. 10.1016/j.parkreldis.2012.01.005 22285756

[ejn15973-bib-0036] Petrucco, C. , Benson, P. , Gordon, L. , Stone, J. , & Johnstone, D. M. (2020). Photobiomodulation as a neuroprotective strategy for Parkinson's disease. In C. R. Martin & V. R. Preedy (Eds.), The neuroscience of Parkinson's disease: Diagnosis and management in Parkinson's disease (pp. 697–712). Elsevier. 10.1016/B978-0-12-815946-0.00040-5

[ejn15973-bib-0037] Potts, L. F. , Wu, H. , Singh, A. , Marcilla, I. , Luquin, M. R. , & Papa, S. M. (2014). Modeling Parkinson's disease in monkeys for translational studies, a critical analysis. Experimental Neurology, 256, 133–143. 10.1016/j.expneurol.2013.09.014 24070854 PMC3962841

[ejn15973-bib-0038] Purushothuman, S. , Nandasena, C. , Johnstone, D. M. , Stone, J. , & Mitrofanis, J. (2013). The impact of near‐infrared light on dopaminergic cell survival in a transgenic mouse model of parkinsonism. Brain Research, 1535, 61–70. 10.1016/j.brainres.2013.08.047 23998985

[ejn15973-bib-0039] Reinhart, F. , Massri, N. E. , Chabrol, C. , Cretallaz, C. , Johnstone, D. M. , Torres, N. , Darlot, F. , Costecalde, T. , Stone, J. , Mitrofanis, J. , Benabid, A. L. , & Moro, C. (2016). Intracranial application of near‐infrared light in a hemi‐parkinsonian rat model: The impact on behavior and cell survival. Journal of Neurosurgery, 124, 1829–1841. 10.3171/2015.5.JNS15735 26613166

[ejn15973-bib-0040] Reinhart, F. , Massri, N. E. , Darlot, F. , Torres, N. , Johnstone, D. M. , Chabrol, C. , Costecalde, T. , Stone, J. , Mitrofanis, J. , Benabid, A. L. , & Moro, C. (2015). 810 nm near‐infrared light offers neuroprotection and improves locomotor activity in MPTP‐treated mice. Neuroscience Research, 92, 86–90. 10.1016/j.neures.2014.11.005 25462595

[ejn15973-bib-0041] Reinhart, F. , Massri, N. E. , Torres, N. , Chabrol, C. , Molet, J. , Johnstone, D. M. , Stone, J. , Benabid, A. L. , Mitrofanis, J. , & Moro, C. (2017). The behavioural and neuroprotective outcomes when 670 nm and 810 nm near infrared light are applied together in MPTP‐treated mice. Neuroscience Research, 117, 42–47. 10.1016/j.neures.2016.11.006 27871905

[ejn15973-bib-0042] Romano, S. , Savva, G. M. , Bedarf, J. R. , Charles, I. G. , Hildebrand, F. , & Narbad, A. (2021). Meta‐analysis of the Parkinson's disease gut microbiome suggests alterations linked to intestinal inflammation. NPJ Parkinson's Disease, 7, 27. 10.1038/s41531-021-00156-z PMC7946946 33692356

[ejn15973-bib-0043] Salehpour, F. , Mahmoudi, J. , Kamari, F. , Sadigh‐Eteghad, S. , Rasta, S. H. , & Hamblin, M. R. (2018). Brain photobiomodulation therapy: A narrative review. Molecular Neurobiology, 55, 6601–6636. 10.1007/s12035-017-0852-4 29327206 PMC6041198

[ejn15973-bib-0044] Santos, L. , Olmo‐Aguado, S. D. , Valenzuela, P. L. , Winge, K. , Iglesias‐Soler, E. , Arguelles‐Luis, J. , Alvarez‐Valle, S. , Parcero‐Iglesias, G. J. , Fernandez‐Martinez, A. , & Lucia, A. (2019). Photobiomodulation in Parkinson's disease: A randomized controlled trial. Brain Stimulation, 12, 810–812. 10.1016/j.brs.2019.02.009 30824206

[ejn15973-bib-0045] Schneider, J. S. , Gonczi, H. , & Decamp, E. (2003). Development of levodopa‐induced dyskinesias in parkinsonian monkeys may depend upon rate of symptom onset and/or duration of symptoms. Brain Research, 990, 38–44. 10.1016/S0006-8993(03)03382-1 14568327

[ejn15973-bib-0046] Shaw, V. E. , Peoples, C. , Spana, S. , Ashkan, K. , Benabid, A. L. , Stone, J. , Baker, G. E. , & Mitrofanis, J. (2012). Patterns of cell activity in the subthalamic region associated with the neuroprotective action of near‐infrared light treatment in MPTP‐treated mice. Parkinsons Disease, 2012, 296875. 10.1155/2012/296875 PMC3361324 22666627

[ejn15973-bib-0047] Shaw, V. E. , Spana, S. , Ashkan, K. , Benabid, A. L. , Stone, J. , Baker, G. E. , & Mitrofanis, J. (2010). Neuroprotection of midbrain dopaminergic cells in MPTP‐treated mice after near‐infrared light treatment. The Journal of Comparative Neurology, 518, 25–40. 10.1002/cne.22207 19882716

[ejn15973-bib-0048] Stone, J. , Johnstone, D. , & Mitrofanis, J. (2013). The helmet experiment in Parkinson's disease: An observation of the mechanism of neuroprotection by near infra‐red light. In E.‐L. Laakso & C. Young (Eds.), Proceedings of the 9th World Association for Laser Therapy Congress (pp. 17–20). Medimond.

[ejn15973-bib-0049] Tuby, H. , Maltz, L. , & Oron, U. (2011). Induction of autologous mesenchymal stem cells in the bone marrow by low‐level laser therapy has profound beneficial effects on the infarcted rat heart. Lasers in Surgery and Medicine, 43, 401–409. 10.1002/lsm.21063 21674545

[ejn15973-bib-0050] Vos, M. , Lovisa, B. , Geens, A. , Morais, V. A. , Wagnieres, G. , van den Bergh, H. , Ginggen, A. , De Strooper, B. , Tardy, Y. , & Verstreken, P. (2013). Near‐infrared 808 nm light boosts complex IV‐dependent respiration and rescues a Parkinson‐related pink1 model. PLoS ONE, 8, e78562. 10.1371/journal.pone.0078562 24244323 PMC3823844

